# MR-Guided Adaptive Radiotherapy for Bladder Cancer

**DOI:** 10.3389/fonc.2021.637591

**Published:** 2021-02-25

**Authors:** Adham Hijab, Boris Tocco, Ian Hanson, Hanneke Meijer, Christina Junker Nyborg, Anders Smedegaard Bertelsen, Robert Jan Smeenk, Gillian Smith, Jeff Michalski, Brian C. Baumann, Shaista Hafeez

**Affiliations:** ^1^ Division of Radiotherapy and Imaging, The Institute of Cancer Research, London, United Kingdom; ^2^ Department of Radiotherapy, The Royal Marsden NHS Foundation Trust, London, United Kingdom; ^3^ Department of Radiation Oncology, Radboud University Medical Center, Nijmegen, Netherlands; ^4^ Department of Oncology, Odense University Hospital, Odense, Denmark; ^5^ Laboratory of Radiation Physics, Odense University Hospital, Odense, Denmark; ^6^ Department of Radiation Oncology, Washington University School of Medicine in St. Louis, St. Louis, MO, United States

**Keywords:** adaptive radiotherapy, bladder cancer, MR guided radiotherapy, MR-linac, MRI

## Abstract

Radiotherapy has an important role in the curative and palliative treatment settings for bladder cancer. As a target for radiotherapy the bladder presents a number of technical challenges. These include poor tumor visualization and the variability in bladder size and position both between and during treatment delivery. Evidence favors the use of magnetic resonance imaging (MRI) as an important means of tumor visualization and local staging. The availability of hybrid systems incorporating both MRI scanning capabilities with the linear accelerator (MR-Linac) offers opportunity for in-room and real-time MRI scanning with ability of plan adaption at each fraction while the patient is on the treatment couch. This has a number of potential advantages for bladder cancer patients. In this article, we examine the technical challenges of bladder radiotherapy and explore how magnetic resonance (MR) guided radiotherapy (MRgRT) could be leveraged with the aim of improving bladder cancer patient outcomes. However, before routine clinical implementation robust evidence base to establish whether MRgRT translates into improved patient outcomes should be ascertained.

## Introduction 

Bladder cancer is the ninth most common cancer diagnosis globally with over 390,000 new cases and over 150,000 deaths occurring each year (1). Muscle invasive bladder cancer (MIBC) makes up approximately 20% of patients at presentation. For these patients, cure is achieved through both effective local treatment and systemic treatment (2, 3).

Radical cystectomy has been the internationally accepted main stay of local treatment for MIBC (4). This requires removal of the bladder, which then necessitates a urinary diversion. Most commonly, this is in the form of an incontinent stoma (ileal conduit). Continent stomas and orthoptic neo-bladder reconstructions are feasible options for some patients. Despite this, continence and sexual function impact significantly on quality of life post-operatively (5–8). A highly selected proportion of patients may be suitable for partial cystectomy by virtue of having a unifocal tumor in a region of the bladder which then permits an adequately safe margin to be obtained without compromise to the bladder capacity. As less than 5% of patients meet these stringent criteria, removal of the whole bladder will be necessary for almost all patients. The clear absence of comparable functional organ substitutes following surgery means that bladder preservation with radiotherapy offers opportunity for cancer cure with organ preservation (3, 9, 10).

Concerns about oncologic equivalence and absence of randomised control data have driven underutilization of radical radiotherapy for the treatment of MIBC (11–13). However, when radiotherapy is used as part of a multi-modality strategy, it achieves similar survival outcomes to radical cystectomy (14, 15). The 5-year cancer-specific survival ranges from 50% to 82% (depending on initial stage), with 5-year overall survival of approximately 50%. Long-term bladder preservation is successfully achieved in up to two-thirds of patients (9). As a result, it would be accepted that patients should be offered opportunity to consider both modalities when either radical treatment would be suitable (3, 10, 16).

The aetiological association of bladder cancer with smoking means patients often have multiple comorbidities on a background of increasing frailty with advancing age that may restrict opportunity for either radical treatment options (17). For these patients, hypofractionated radiotherapy offers prospect for long-term disease and symptom control (18, 19).

In both the radical and palliative bladder radiotherapy settings, there remains opportunity to improve clinical outcomes further by overcoming some of the challenges that bladder radiotherapy poses. In this article, we examine the technical challenges of bladder radiotherapy and explore how magnetic resonance (MR) guided radiotherapy (MRgRT) could provide a solution for geometric and biologically adapted treatment delivery.

## Current Role of MR Imaging in Bladder Cancer

The tumor staging of bladder cancer is contingent on accurately determining the presence of muscle invasion. Given the different treatment approaches for NMIBC and MIBC, establishing the correct tumor stage is critical in deciding the correct treatment strategy (3, 20). Although CT provides high spatial resolution allowing visualization of extra-vesical spread, it is not a reliable means of determining the extent of muscle involvement (21). It is limited both by inter-observer variability and inability to distinguish the muscle layers of the bladder (22, 23). As a result, the current standard means of diagnosing and staging MIBC remains performing a TURBT with the aim of ensuring bladder muscle is included in the specimen so that its involvement can be ascertained (3, 20, 24). However, TURBT remains imperfect as it risks under staging in 25%–50% of patients (25–27).

Magnetic resonance imaging (MRI) staging accuracy exceeds those reported for TURBT in terms of distinguishing between MIBC (≥T2) and NMIBC (≤T1) (28, 29). Three meta-analyses have evaluated the performance characteristics of multi-parametric MRI (mpMRI) for local tumor staging across approximately 5,000 patients. These studies reported similar results, with pooled sensitivity of 0.87 (95% Confidence interval, CI 0.82–0.91), 0.90 (95% CI 0.83–0.94), and 0.92 (95% CI 0.88–0.95), and specificity of 0.79 (95% CI 0.72–0.85), 0.87 (95% CI 0.78–0.93), and 0.88 (95% CI 0.77–0.94) (28–30).

**Figure 1:**
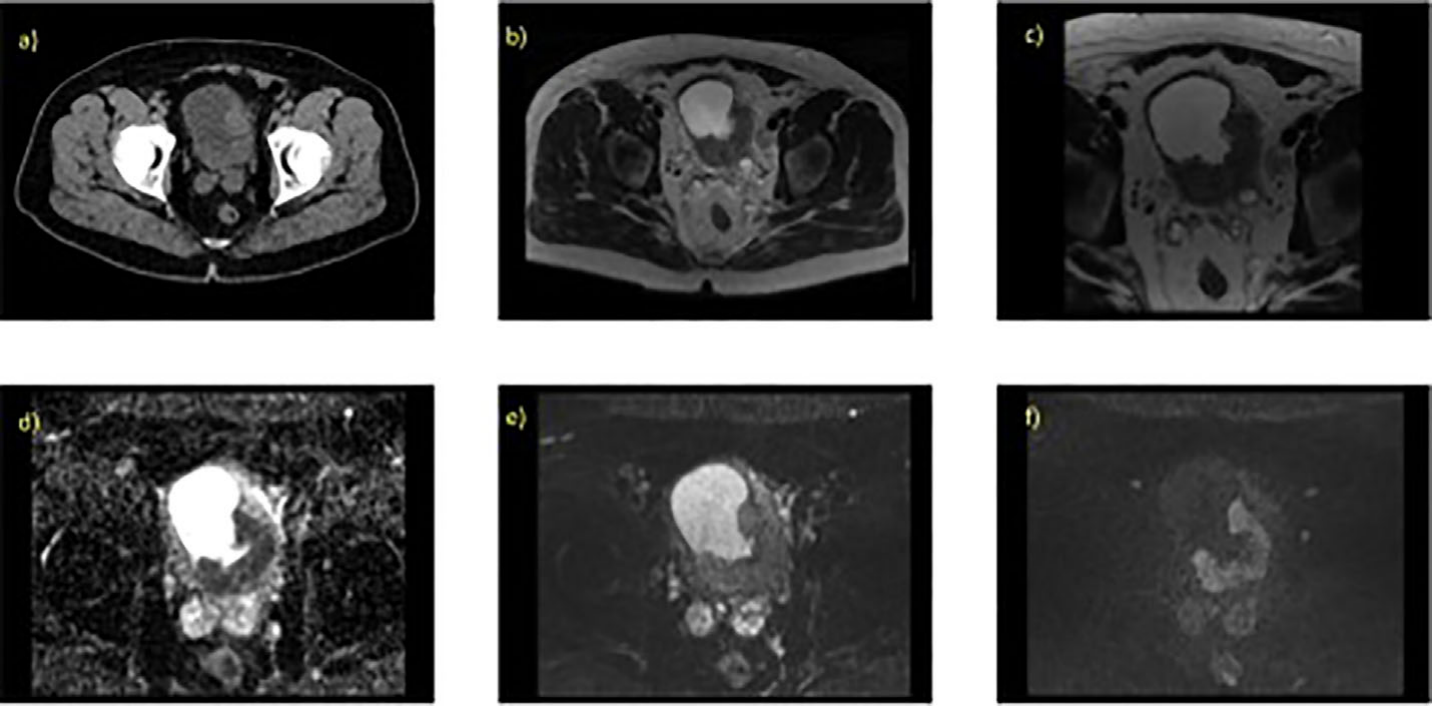
Localized MIBC as evaluated on T2W and DWI with the associated parameter settings for 1.5T MRI. 70 year old male with known T3 N0 M0 bladder cancer, tumour is present at the left ureteric orifice (extending posteriorlaterally) **(A)** contrast enhanced CT scan, axial slice through pelvis, **(B)** axial T2W (large field of view) showing hypo intense lesion, **(C)** axial T2W small field of view **(D)** corresponding ADC map, **(E)** axial DWI at b-value 0, **(F)** axial DWI at b-value 750.

A mpMRI examination for bladder cancer staging usually consists of a T2-weighted image (T2W) with diffusion-weighted image (DWI), or dynamic contrast enhancement (DCE) image (28–31). There is suggestion however that mpMRI using DWI is the optimal protocol for tumor staging of bladder cancer (29, 30). **Figure 1** illustrates example image of a localized MIBC as evaluated on 1.5T MRI.

In order to standardize the image acquisition, interpretation, and reporting of mpMRI for newly diagnosed bladder cancer, the Vesical Imaging-Reporting and Data System (VI-RADS) was developed in 2018 (31). This is a five-point qualitative scoring system of bladder tumors as seen on T2W, DWI, and DCE imaging, to determine the likelihood of muscle invasion. The final score is based on T2W imaging because of its high spatial resolution to evaluate the integrity of the muscle layer. Definitive muscular invasion is decided by the assessment of DWI and DCE. However, as DWI improves the accuracy of distinguishing MIBC, it is relied upon particularly when there is discordant scoring between T2W and DCE sequences (29, 31–33).

Multi-institutional studies applying VI-RADs scoring (1-5) to mpMRI interpretation to determine local staging demonstrates high sensitivity and specificity when a cut off score of ≥3 is used to describe likelihood muscle invasion (34–38). VI-RADS scoring also reflects good to excellent interobserver reporting agreement, with indices of agreement ranging between 0.73 and 0.92 (34–37). Despite this evidence, mpMRI has not yet established its place as recommended and preferred standard imaging for local bladder cancer staging in clinical guidance (3).

In prostate cancer mpMRI has been shown to identify those men who could safely avoid unnecessary biopsy with the aim of enabling detection of clinically significant disease (39). In bladder cancer, it is also hypothesized that mpMRI may also serve as a triage test prior to TURBT (40). The advantage this presents for MIBC patients is that it would potentially reduce delays to definitive treatment, avoids under staging on initial TURBT, and minimizes the risk of systemic circulating cancer cell dissemination occurring as a result of bladder perforation with TURBT (25, 26, 41, 42). The possibility that the TURBT may be completely avoided when suspicion of MIBC is high on mpMRI is being explored in a randomized phase 2/3 trial (BladderPath, ISRCTN reference number 35296862) (43). This trial aims to compare the standard diagnostic pathway consisting of flexible cystoscopy and biopsy, with imaging followed by TURBT versus a risk stratified imaging directed pathway whereby if on flexible cystoscopy there is clinical suspicion of possible MIBC, a biopsy is taken and patients proceed to mpMRI. If the mpMRI supports likelihood of NMIBC, patients then proceed to TURBT otherwise if the mpMRI supports likelihood of MIBC patients proceed to directly to treatment. Initial feasibility to randomize possible MIBC patients to a TURBT directed diagnostic pathway or mpMRI directed diagnostic pathway has been successfully demonstrated. The trial is ongoing to investigate how a mpMRI-driven diagnostic pathway impacts on time to correct therapy for MIBC and NMIBC and clinical progression-free survival (43).

## Rationale for MR-Guided Online Adaptive Bladder Radiotherapy

### MRI Improves Target Visualization

The uncertainties of using CT for bladder tumor staging also impact on the ability to reliably define the outer bladder wall and gross tumor volume (GTV) within the bladder. As a result, use of CT leads to significant inter-observer target delineation variability particularly at interfaces with neighboring structures such as small bowel or prostate, and in the presence of extra-vesical spread (44–47). Poor target delineation is a major source of systematic inaccuracies in radiotherapy (45). The improved soft tissue contrast of MRI may help address this.

The GTV visibility in bladder cancer however can be hampered after TURBT and good response to neo-adjuvant chemotherapy (46). Insertion of radio-opaque markers at cystoscopy to demarcate the visible tumor extension has been explored (48). Surgical clips or gold fiducial markers can be inserted at the borders of visible tumor or tumor bed *via* cystoscope (49–51). Although they provide excellent visualization on CT, these markers are prone to migration and fall out in up to 50% of cases following implantation (49, 52). Diathermy post insertion or gold seeds with micro-tines further improve retention rates but net marker losses (up to 18%) are still seen (50, 51). Metallic fiducial markers do not yield a signal on MRI and appear dark. By using T2*weighted sequences, the signal loss can be emphasized such that their position can be identified allowing them to be used to guide localization on MRI (53).

Iodized oil contrast (Lipiodol^®^), 0.25–0.50cc injected sub-epithelially into the bladder wall has also been used as an alternative fiducial marker (54–57). Its use is limited to patients with no history of contrast medium sensitivity or active thyroid disease (54, 58). It is not subject to the same frequency of marker loss, but the liquid nature of the contrast medium means intra- and extra-vesical spillage can occur (54–56). In circumstances of high concentration, this can lead to streak artefacts on CT (59, 60). Lipiodol is not visible on MRI.

Novel radiographic gel-like markers (BioXmark®) that are liquid, with low initial viscosity prior to and during injection but transforms into a highly viscous liquid to form a 3D gel-like shape have also been investigated (61). It produces signals void on MRI in phantom studies (62). Further work is in progress to assess this marker when used clinically for bladder MRI evaluation.

### Adaptive Radiotherapy to Address Target Motion

The bladder is relatively mobile target subject to filling variation and deformation. It is fixed at the caudal pole and is abutted by the rectum or uterus posteriorly. Therefore, as the bladder volume increases non-uniform organ expansion generally occurs which is more pronounced in the cranial and anterior directions (47, 63–66). The magnitude of this change is rarely consistent or predictable (67, 68). Patient interventions such as drinking protocols, catheterization, dietary modifications, and laxatives have been explored but do not consistently reduce bladder target variation (60, 69, 70).

#### Inter-Fractional Motion Mitigation

In an attempt to compensate for both the variability of the bladder shape, and size between treatments (inter-fraction), historically large population-based margins (up to 1.5–2cm) have been applied to create the planning target volume (PTV). Despite the use of such large margins to address the bladder target positional uncertainties, without the adoption of soft tissue image guidance, geographical misses will occur at treatment delivery (71).

Pre-treatment, in-room three-dimensional volumetric soft tissue imaging provides anatomical information that can feedback into the plan and adapt dose delivery optimization (72). The overall aim of these adaptive radiotherapy strategies is to further improve the fidelity of dose delivered to target in order to reliably reduce the PTV so dose to normal tissues can also be reduced. In bladder cancer radiotherapy, two main adaptive approaches based on the wide availably of cone bean CT (CBCT) have seen drift into clinical practice based on reported dosimetric gains (68, 73).

The composite volume method is an offline adaptive radiotherapy approach that utilizes information from the verification CBCT acquired for the first 3–5 fractions to determine a patient specific internal target volume (ITV) informed by the maximal excursions of the bladder actually occurring. A smaller margin to account for remaining residual uncertainties is then applied to create a new PTV and plan. This solution adequately maintains bladder target coverage and reduces the PTV by approximately 40%–50% compared to population based PTV approach (74, 75). The main disadvantage is that patients can only benefit from the adaptive radiotherapy strategy after sufficient number of verification CBCTS images has been acquired. This presents limitations in its application to hypofractionated regimes because a significant proportion of treatment course would already have been delivered before a new plan can be created.

The alternative and more widely adopted method currently employed is to generate a library of patient specific treatment plans with varying PTV sizes (73). Using the CBCT acquired prior to each fraction, the anatomy is assessed to select the most appropriate plan that covers the bladder target with minimal normal tissue exposure. The library of plans can be created by applying either variable margins or by modeling the patient’s own bladder filling pattern using either serial planning CT scans or the verification CBCTs from the initial fractions (76–78). This solution also successfully maintains target coverage, and reduces the PTV by approximately 40% with subsequent reduction in normal tissue irradiation (79). The main disadvantage is that a discrete library created to cover the spectrum of interfraction variation means the individual conformity of the selected plan to the imaged bladder on the day can be relatively poor (80). It is also possible in some circumstances that none of the plans in the library encompass the imaged bladder target on the day (78, 80).

Modeled approaches in bladder cancer radiotherapy illustrate that by adopting an online replanning adaptive radiotherapy process, whereby the patient’s treatment plan is produced based on the actual anatomy seen while they are on the treatment couch would further improve target coverage and OAR sparing (81, 82). In work comparing standard single plan, with different adaptive strategies the volume of normal tissue receiving more than 95% of the prescribed dose was reduced to 66% (range 48%–100%) with library approach and to 41% (range 33%–50%) with daily re-optimization (81). Considerable normal tissue sparing potential therefore exists for bladder cancer patients with online re-optimization.

The availability of hybrid systems that incorporate both MRI scanning capabilities and linear accelerator (MR-Linac) allows an in-room, real-time MRI scan to be obtained immediately prior each fraction (83–85). As MRI yields superior soft-tissue contrast compared to CBCT it would be preferred means for accurate bladder target delineation and organs at risk (OARs), i.e., rectum and bowel identification to inform re-optimization at each fraction (86). Feasibility of these platforms to deliver an MR-informed fully online re-optimized new bladder plan at each fraction has been demonstrated (87, 88).

#### Intra-Fractional Motion Mitigation

Stochastic variation in the organ filling, deformation, and peristaltic motion means that changes will occur in the bladder target and OARs within the time scale of pre-treatment imaging and delivery of each individual treatment fraction. This necessitates additional consideration to determine the best means of accommodating for this motion in order to minimize risk of geographical miss.

The most common strategy in bladder cancer radiotherapy is to treat on an empty bladder and passively manage intra-fractional change by the application of a margin that will encompass the magnitude of motion likely to occur within the time frame of the workflow. For treatment delivery based on the CBCT adaptive solutions described above, intra-fraction margins ranging from 2 mm to 7 mm have been suggested (76, 77, 79, 81, 89, 90). This margin may also be influenced by treatment technique, as intensity modulated arc therapy (IMAT) is associated with faster delivery times than fixed field IMRT so facilitating use of smaller intra-fraction margins (91).

In a patient population who had serial MRI scans acquired at 2 minute intervals for up to 10-min post voiding, it was possible to demonstrate that the application of anisotropic margins (14 mm cranially and anteriorly, 9 mm posteriorly, and 5 mm in all other directions) successfully maintained target coverage as evaluated on the 10-min MRI scan for the entire treatment course (82). Target under dosing (≥D1cc <95% of the prescribed dose) was seen in 4% of fractions compared to 20% when a 5 mm isotropic margin was used (82).

Currently, treatment workflow times for utilization of an MRgRT online reoptimization approach are in the region of approximately 30–40 min (87, 92, 93). It has been successfully shown that an anisotropic margin of 15 mm applied cranially and anteriorly, 1 cm posteriorly, and 5 mm in all other directions will successfully maintain target coverage in 96.6% of fractions as assessed on the post treatment MRI scan (87). The mean conformity of the 95% isodose to the post treatment bladder target is 2.4 (range 1.5–3.6), suggesting the intra-fraction margin could be reliably reduced in some instances (87). While maintenance of target coverage throughout the fraction delivery is a priority, the potential gains of online re-optimization would be mitigated by the use of over-generous intra-fraction margins.

The alternative approach is to actively manage intra-fraction change with MR guided motion management. During beam on period, continuous MR imaging can be acquired for real-time motion monitoring, tracking, and or gating. A tracking slice is positioned to include a cross-sectional axis at the target volume of interest. A minimum tracking boundary or motion monitoring structure is set such that if a pre-specified proportion of the tracked target leaves this boundary, the beam will turn off (10). This allows extremes of anatomical changes to be detected while the target is being irradiated to minimize the risk of a geographical miss (10, 94).

MR guided tumor tracking has been successfully used on the MR-Linac for treatment of tumors of the upper abdomen and prostate (95–97). However, the challenge this presents for bladder cancer radiotherapy is that tracking alone is not necessarily a universal solution if the target is increasing in overall volume as occurs with whole bladder radiotherapy (65, 66). It raises the question then, could the tumor itself be tracked and could this region be safely prioritized over the uninvolved bladder.

### Enabling Tumor-Focused Partial Bladder Irradiation

Tumor-focused partial bladder radiotherapy is attractive for two main clinical reasons: firstly, the reduced high dose opens the possibility that treatment-related toxicity could be reduced; and secondly, it opens the possibility for dose escalation to the tumor beyond limits currently determined by the whole bladder tolerance of 64-65Gy in 2Gy per fraction (98–100).

Whole bladder radiotherapy has been the accepted convention even in the presence of unifocal disease possibly because of the difficulty in identifying the tumor within the bladder on CT and the historical inaccuracies of treatment delivery described above. Nevertheless, evidence to date supports that partial bladder irradiation is likely to be safe (3, 101–103).

Bladder brachytherapy has been used for a highly select patient population with unifocal small lesions (≤50 mm) achieving similar outcomes to a matched population undergoing radical cystectomy (104). It is not widely accepted or recommended as an organ-conserving treatment option mainly because technical expertise is confined to highly specialized centers and no randomized control data is available (3, 101).

Randomized control trials of whole bladder versus tumor-focused partial bladder external beam radiotherapy have successfully demonstrated that tumor-focused partial bladder radiotherapy could be utilized with no adverse effect on local control (103, 105). However, these randomized controlled trials failed to show decrease treatment related toxicity (103, 105). A number of technical aspects are likely to have mitigated any benefit from a reduced high dose volume. Treatment was planned and delivered on an empty bladder. In addition, delineation of the tumor within the bladder using a planning CT scan would have invariably led to overestimating the GTV size (44–46). The subsequent isotropic 1.5 cm expansion margin around the GTV to generate the PTV boost volume from which a 3D conformal treatment plan was created would then leave very little additional normal tissue sparing compared to whole bladder treatment. Setup in the era of these trials was either to skin or bone and preceded soft tissue verification, so it can be assumed that with 1.5 cm margin target coverage may have only been approximately 60% (71). Dose unsuccessfully delivered to target would have resulted in unwanted normal tissue irradiation.

Many investigators have sought to overcome these challenges by using library of plans to deliver tumor-focused high dose radiotherapy on filled or partially filled bladder (80, 106, 107). The advantage of striving for a fuller bladder in these circumstances is that it reduces dose to the uninvolved bladder and provides greater opportunity for normal bladder sparing. Treatment delivered in these trials used either fixed field IMRT or IMAT. This improves conformity of radiation fields around the target volume, relative to 3D conformal techniques (91). In comparisons of clinical outcomes of bladder cancer radiotherapy, IMRT has been reported to significantly reduce acute CTCAE grade ≥2 diarrhoea compared to 3D conformal radiotherapy (56% versus 30%; p = 0.008) (108). Whether using library of plans to escalate tumor-focused dose translates into clinically meaningful outcomes will be evaluated in an international randomised phase II trial (RAIDER, NCT02447549) (109).

Dosimetric analysis of library of plans to deliver tumor-focused high dose radiotherapy reveals that although excellent target coverage can be achieved meeting normal bladder and bowel constraints, the high mean conformity of the 95% isodose of the selected plan to the tumor boost as seen on CBCT is 5.0 (SD 2.2, range 2.1–21.4) and the whole bladder is 3.5 (SD 1.0, range 1.7–8.9). This suggests large volume non-target irradiation is still occurring (80). The MR-Linac may therefore open opportunity for an online re-optimized tumor-focused partial bladder approach.

**Figure 2:**
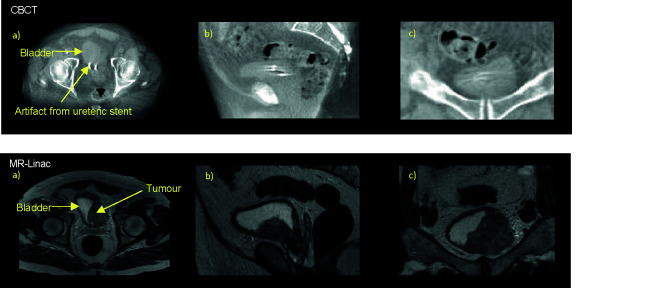
Online pre-treatment CBCT and MR (T2W) images. Bladder tumour at left bladder wall as seen on axial a), sagittal b), and coronal c) views of the pelvis on corresponding CBCT and T2W taken on the MR-Linac, here urine appears bright and tumour dark/hypointense.

Successful tumor-focused partial bladder irradiation is dependent on ability to define GTV on both the planning CT and CBCT. Although CBCT allows reasonable discrimination of the bladder wall, visualization of the tumor itself is challenging (80, 110). As local recurrences occur most frequently at the original MIBC tumor site, correctly identifying the GTV becomes increasing critical particularly in the era of margin reduction (111). The superior soft tissue contrast of MRI may therefore enable more reliable tumor-focused partial bladder radiotherapy (**Figure 2**).

The MR-Linac may also provide greater opportunity to assess how the tumor moves in relation to the filling status of the bladder to determine the most appropriate intra-fraction margins for partial bladder radiotherapy. Work to date suggests that the bladder tumor is relatively rigid and non-elastic compared to non-tumor-bearing bladder regions but this is based on CT interpretation (112).

## Workflow Considerations for Bladder Treatment on the MR-Linac

**Figure 3:**
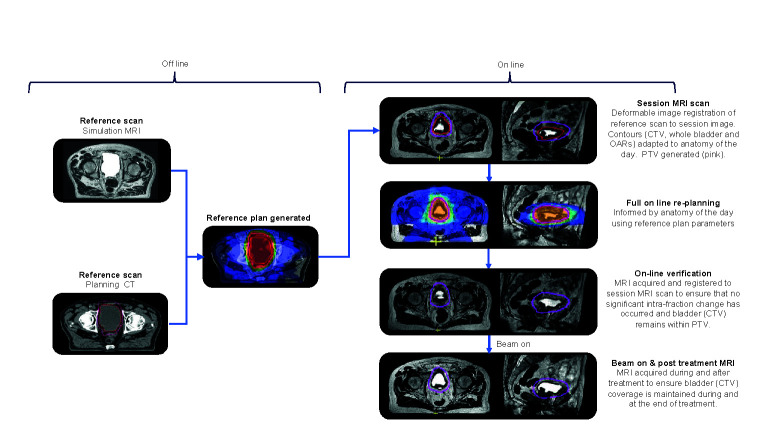
Overview of the principal workflow components of online reoptimization using MRgRT.

An overview of the principal workflow components is presented in **Figure 3**. For treatment of the whole bladder on the MR-Linac, workflow time pressures are critical because of the anticipated intra-fraction volume increase. If workflow time could be reduced, the margins currently applied to accommodate for this change could also be reduced. Several considerations can assist with achieving this.

Ideally as little time as possible should be spent re-optimizing the daily treatment plan. This can be aided by generating a robust planning class solution from the outset to minimize the need for online modification and experimentation. This should be robust to the expected daily changes in anatomy that will occur.

Prior to starting treatment, a reference plan is created. A planning CT (CT_planning_) and, or a simulation MR (MR_planning_) scan is acquired with an empty bladder. This is achieved by asking the patient to void immediately prior to scanning. The CT_planning_ is used for density information and it is deformably registered to the MR_planning_. It is also possible that at simulation serial images over time are acquired to estimate a “patient specific” intra-fraction bladder filling PTV margin.

When patients attend for treatment, they are asked to void their bladder immediately prior to set up. A session or pre-treatment MRI (MR_session_) image is acquired on the MR-Linac which is registered to the planning reference image (CT_planning_ or MR_planning_). The contours from the planning reference image are propagated to the MR_session_ image using deformable registration or segmented using artificial intelligence contouring algorithms (113). The contours are reviewed and corrected if necessary. To speed up the outlining time, more accurate delineation of OARs is limited to a 2 cm region around the target. The consequence of having less accurate contours is that, although the dose distribution will still be close to optimal, the reported dose statistics for these OARs will be less reflective of actual dose to these structures. This trade-off is made to balance the desire for accurate delineation and the fact that the OARs underlying those contours are continuously changing whilst they are being delineated.

A new plan with full re-optimization is created. For online bladder planning dose-volume metrics do not have to be used, instead focus can be placed on how rapidly the dose falls off away from the target. Here, the optimizer only considers the dose gradient in the region where the OAR abuts the target, and as such is not dependent on the overall OAR volume. This approach is also less sensitive to accurate delineation of the OARs, as only the approximate region where they border the target is needed.

During the optimization process, a fast T2W MRI (MR_verfication_) is acquired to confirm that appropriate target coverage is maintained either by reviewing the PTV coverage of the bladder or the isodose coverage of the bladder. If the bladder is not optimally covered then the plan can be shifted relative to the isocenter and dose recalculated on the MR_session_ (114, 115). If this maneuver would also not sufficiently cover the bladder target then it would be recommended that the patient is removed from the couch, voids their bladder, and are treated with the reference plan. Prior to the subsequent fraction patient factors contributing to rapid bladder filling, i.e., pre-treatment diuretic or excessive hydration should be explored and managed. It may also be necessary to review and increase the intra-fraction margin.

At treatment delivery, cine MR can be used to monitor bladder motion during beam on with the option that should the bladder move out of the PTV or the pre-defined motion monitoring structure, the treatment can be interrupted if required. A post treatment T2W MRI (MR_post_) is acquired immediately following delivery for offline dose assessment of the treatment delivered. The difference between planned dose on MR_session_ and delivered dose as determined on MR_post_ could potentially be incorporated into the online adaption strategy and compensated for at the subsequent fractions, if clinically indicated.

Currently the time to deliver this workflow at best is between 15 and 27 min (personal communication, A Bertelsen & C Nyborg, Odense University Hospital, Denmark) but we have found the median total time for patients on the treatment couch is 39 min (range 33–48) (87). We expect that this will be reduced further with faster image acquisition, improvements in auto-contouring, increased computational ability for plan optimization and dose calculation, and the implementation of IMAT delivery techniques.

## Beyond Geometric Adaption

MRI could be used to acquire biological information about the bladder tumor. This could provide opportunity to develop MRI informed biologically adapted radiotherapy approaches (116).

DWI is a functional imaging technique dependent on the inhibitory effect of cell membranes to the random motion of water molecules. The higher cellular density of tumors compared to normal tissue means they demonstrate higher signal intensity, i.e., restricted diffusion on MRI, reflected quantitatively in a low mean apparent diffusion coefficient value (ADC). Per pixel ADC throughout the tumor volume can be used to capture the regional heterogeneity known to exist within tumors which may have prognostic and predictive value (117–121). As the local relapse site following radiotherapy is at the site of the MIBC tumor, it is hypothesized that by escalating dose to the tumor region of highest cellularity, local control rates could be improved (111).

Following successful treatment, the ADC value increases, reflecting decrease in cellularity. In MIBC ADC change is an independent predictor of pathological response (122, 123). Given serial DWI acquisition on the MR-Linac is possible at each fraction, there is potential for monitoring ADC change throughout treatment with identification of early non-responders who may benefit from change in treatment approach (124). As such MRI offers opportunity for a response adapted radiotherapy delivery.

Tumor hypoxia in MIBC is a potential predictor of radiotherapy response with effective modification improving outcome (125, 126). MRI can be used to measure and map tumor hypoxia in a number of ways not otherwise possible on biopsy or serum surrogates (127, 128). Intrinsic susceptibility weighted or blood oxygenation level dependent MRI (BOLD), exploits the difference in magnetic susceptibility of oxyhaemoglobin and deoxyhaemoglobin to generate contrast and identify regions of hypoxia (129).

Visualization of tumor blood flow can be used as a surrogate to identify areas of hypoxia. DCE enables *in vivo* assessment of tumor blood flow and permeability using paramagnetic contrast agents. DCE has been shown to have ability to predict treatment response in MIBC following chemotherapy (130). Experimental models demonstrate the potential effectiveness of hypoxia informed boost dose delivery to increase tumor control (126). Future partial bladder radiotherapy approaches could therefore inform a mpMRI derived biological target volume. Given this volume is up to 45% smaller than an anatomically defined bladder GTV, it opens the possibility of further normal tissue sparing (131). As the volume of radiation influences the immunogenic potential of the tumor microenvironment, defining alternative meaningful target sub-volumes particularly with systemic immunotherapy warrants further evaluation (132, 133).

## Conclusion

MRgRT heralds a paradigm shift for bladder cancer patients with potential gains to be had at the simulation, treatment delivery, and response assessment stages. Whether the closer integration of MRI into the bladder patient radiotherapy pathway translates into clinical gains for our patient population is still yet to be determined. A framework for clinical evaluation of MR-Linac technologies has been suggested (134, 135). We would strongly advocate participation in clinical trials to generate robust evidence base to prove our expectations (and hopes) of further improving bladder cancer patient outcomes with MRgRT.

## Author Contributions

All authors meet at least of one the criteria recommended by the ICMJE. SH wrote the first manuscript draft. AH, BT, AB, IH, HM, CN, RS, and GS were all sub-section contributors. All authors contributed to the article and approved the submitted version.

## Conflict of Interest

BCB reports personal fees from Mevion, personal fees from Mevion/Sanofi, outside the submitted work. SH reports non-financial support from Elekta (Elekta AB, Stockholm, Sweden), non-financial support from Merck Sharp & Dohme (MSD), personal fees and non-financial support from Roche outside the submitted work.

The remaining authors declare that the research was conducted in the absence of any commercial or financial relationships that could be construed as a potential conflict of interest.
